# High pericoronary adipose tissue attenuation on computed tomography angiography predicts cardiovascular events in patients with type 2 diabetes mellitus: post-hoc analysis from a prospective cohort study

**DOI:** 10.1186/s12933-022-01478-9

**Published:** 2022-03-18

**Authors:** Keishi Ichikawa, Toru Miyoshi, Kazuhiro Osawa, Mitsutaka Nakashima, Takashi Miki, Takahiro Nishihara, Hironobu Toda, Masatoki Yoshida, Hiroshi Ito

**Affiliations:** 1 Department of Cardiovascular Medicine, Okayama University Graduate School of Medicine, Dentistry and Pharmaceutical Sciences, 2-5-1 Shikata-cho, Kita-ku, Okayama, 700-8558 Japan; 2 Department of General Internal Medicine 3, Kawasaki Medical School General Medicine Centre, Okayama, Japan

**Keywords:** Diabetes mellitus, Coronary computed tomography angiography, Perivascular coronary inflammation

## Abstract

**Background:**

Pericoronary adipose tissue (PCAT) attenuation on coronary computed tomography angiography (CTA) is a non-invasive biomarker for pericoronary inflammation. We aimed to investigate the prognostic value of PCAT attenuation in patients with type 2 diabetes mellitus (T2DM).

**Methods:**

We included 333 T2DM patients (mean age, 66 years; male patients, 211; mean body mass index, 25 kg/m^2^) who underwent clinically indicated coronary CTA and examined their CT findings, coronary artery calcium score, pericardial fat volume, stenosis (> 50% luminal narrowing), high-risk plaque features of low-attenuation plaque and/or positive remodelling and/or spotty calcification, and PCAT attenuation. We assessed PCAT attenuation in Hounsfield units (HU) of proximal 40-mm segments of the left anterior descending artery (LAD) and right coronary artery (RCA). Cardiovascular events were defined as cardiac death, hospitalisation for acute coronary syndrome, late coronary revascularisation, and hospitalisation for heart failure.

**Results:**

During a median follow-up of 4.0 years, we observed 31 cardiovascular events. LAD-PCAT attenuation was significantly higher in patients with cardiovascular events than in those without (− 68.5 ± 6.5 HU vs − 70.8 ± 6.1 HU, p = 0.045), whereas RCA-PCAT attenuation was not (p = 0.089). High LAD-PCAT attenuation (> − 70.7 HU; median value) was significantly associated with cardiovascular events in a model that included adverse CTA findings, such as significant stenosis and/or high-risk plaque (hazard ratio; 2.69, 95% confidence interval; 1.17–0.20, p = 0.020). After adding LAD-PCAT attenuation to the adverse CTA findings, the C-statistic and global chi-square values increased significantly from 0.65 to 0.70 (p = 0.037) and 10.9–15.0 (p = 0.043), respectively.

**Conclusions:**

In T2DM patients undergoing clinically indicated coronary CTA, high LAD-PCAT attenuation could significantly predict cardiovascular events. This suggests that assessing LAD-PCAT attenuation can help physicians identify high-risk T2DM patients.

**Supplementary Information:**

The online version contains supplementary material available at 10.1186/s12933-022-01478-9.

## Background

Coronary artery disease (CAD) is the leading cause of mortality in patients with type 2 diabetes mellitus (T2DM) [1, 2]. With the increasing global prevalence of T2DM, preventing CAD in T2DM patients is gaining importance as a public health measure [3]. Coronary computed tomography angiography (CTA) is a useful diagnostic modality to evaluate CAD in T2DM patients [4, 5]. Atheromatous plaques that show positive remodelling and low plaque density during coronary CTA evaluations are associated with a higher risk of future cardiovascular events [6, 7]; however, in general, the prognostic value of these CTA-verified plaque features to predict cardiovascular events is limited [8]. Thus, a diagnostic approach to help identify T2DM patients vulnerable to cardiovascular events is needed.

Recently, change in pericoronary adipose tissue (PCAT) attenuation, assessed on coronary CTA, was introduced as a novel indicator of inflammation [9]. In the Cardiovascular Risk Prediction using Computed Tomography (CRISP-CT) study, high PCAT attenuation around the left anterior descending artery (LAD) and right coronary artery (RCA) was shown to be a significant risk factor for increased cardiac mortality [10]. Furthermore, PCAT attenuation assessment has led to a significant improvement in cardiovascular risk and predicted risk better than the characteristics of coronary CTA alone [10, 11]. Thus, PCAT attenuation has been identified as a novel marker for cardiovascular risk. Although PCAT attenuation has the potential to enhance clinical risk assessment, few studies have investigated the prognostic value of PCAT attenuation to date. Therefore, further studies including a larger study population, including patients with T2DM, are warranted to confirm the clinical significance of PCAT attenuation.

Herein, we hypothesised that assessing PCAT attenuation on coronary CTA can help predict the risk of cardiovascular events in T2DM patients with suspected CAD.

## Methods

### Study population

**Fig. 1:**
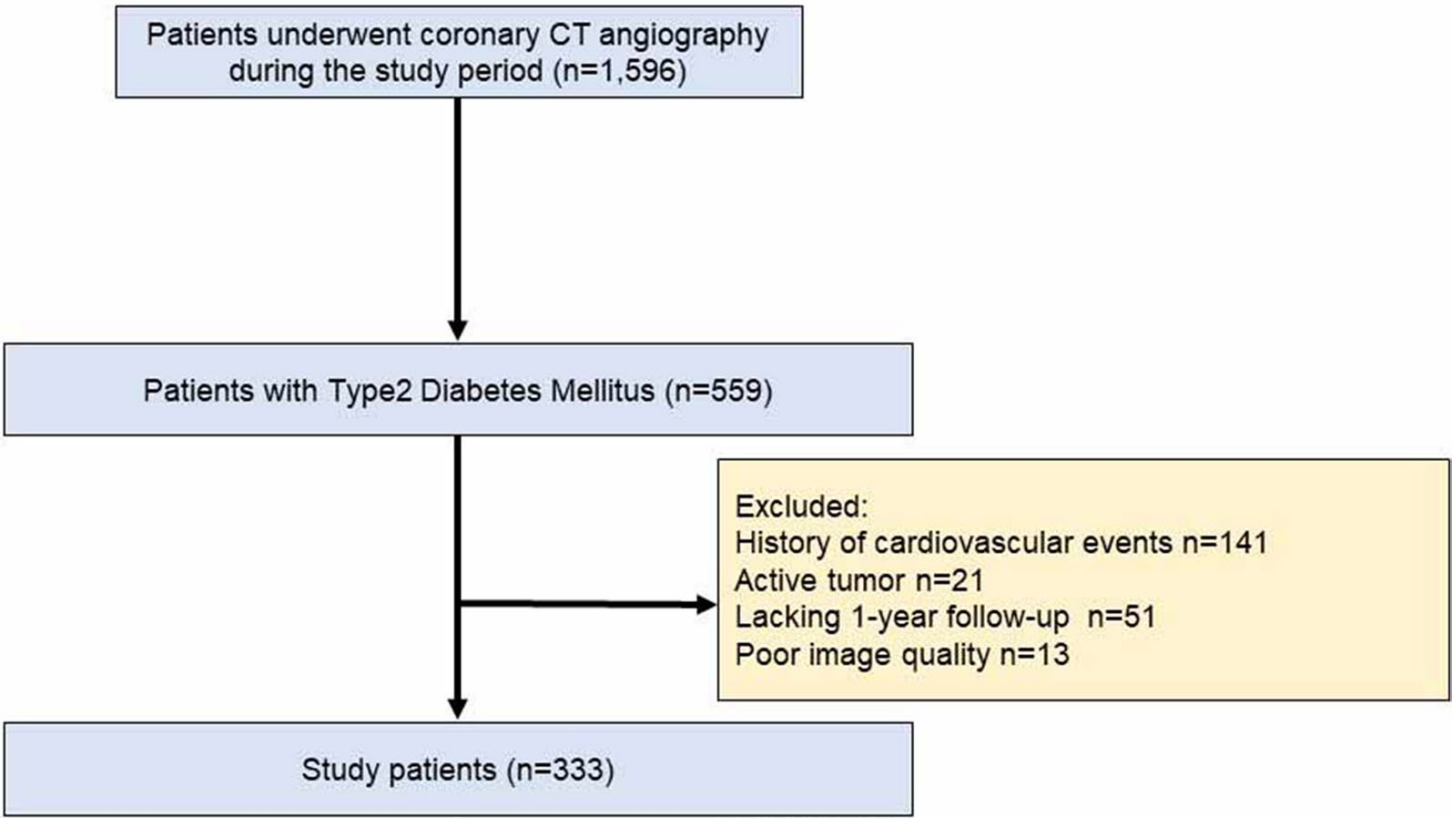
Flowchart showing the study design

The current study population was derived from a prospective, single centre, cohort study comprising Japanese patients who underwent coronary CTA at Okayama University. A study including some patients in this cohort has already been published [12]. We prospectively gathered data on the clinical features, coronary CTA findings, and patient outcomes. In this study, post hoc analysis was used for PCAT attenuation measurement. Of 1596 Japanese outpatients without a history of CAD who underwent coronary CTA for suspected stable CAD from August 2011 to December 2016, the following were excluded: (1) serum creatinine ≥ 1.5 mg/dL; (2) allergy to contrast media; and (3) pregnancy or being of childbearing age and not using contraceptives. Based on these criteria, we enrolled 559 T2DM patients who underwent clinically indicated coronary CTA for suspected CAD. Of these, we excluded patients with a history of cardiovascular events (n = 141), a coexisting active tumour (n = 21), < 1 year of follow-up (n = 51), and poor image quality (n = 13). Finally, we included 333 T2DM patients in the final analysis (Fig. 1). T2DM was defined according to the American Diabetes Association guidelines [13].

At baseline, we used standardised questionnaires to collect information on patients’ medical history, medication use, and smoking status. The study protocol was approved by the institutional review board of the Okayama University Graduate School of Medicine. The study procedures were conducted following the principles of the Declaration of Helsinki, and all the enrolled patients provided written informed consent to participate.

### Outcome data

We reviewed medical records and conducted telephone interviews to collect follow-up information. Cardiovascular events included cardiac death, hospitalisation for acute coronary syndrome, late coronary revascularisation, and hospitalisation for heart failure. We classified all deaths as cardiac, non-cardiac, or unknown. For our analysis, unknown deaths were not counted as cardiovascular events. Acute coronary syndrome collectively included myocardial infarction and unstable angina. We defined late coronary revascularisation as a percutaneous coronary intervention or coronary artery bypass grafting to treat stable CAD that showed a new positive functional test for ischaemia > 90 days after coronary CTA. We excluded cardiovascular events that occurred in patients with revascularisation scheduled within 90 days on indexed coronary CTA findings from our analysis; instead, we censored these patients at the time of their first revascularisation to eliminate confounding.

### Assessment of risk factors

Hypertension was defined as seated blood pressure > 140/90 mmHg or current treatment with antihypertensive medication. Dyslipidaemia was defined as one or more of the following: serum triglycerides ≥ 150 mg/dL, high-density lipoprotein cholesterol < 40 mg/dL, low-density lipoprotein cholesterol ≥ 140 mg/dL, or current treatment with a lipid-lowering drug. Smoking status was defined as currently smoking or not smoking. We calculated the Suita score, widely used to estimate the risk of coronary heart disease in the Japanese population, for all patients [14]. The Suita score accounts for age, sex, smoking status, T2DM, blood pressure, low-density lipoprotein (LDL)-cholesterol, high-density lipoprotein cholesterol, and renal function. The Suita score is used to identify individuals categorically as low (score ≤ 40), intermediate (score 41–55) or high risk (score ≥ 56) for coronary heart disease.

### Acquisition and analyses of coronary CTA

All patients arrived at the hospital 1 h before the scheduled CT time and mandatorily received a dose of oral short-acting nitroglycerin. If their heart rate was > 70 beats/min, an oral or intravenous β-blocker was administered to reduce the heart rate further. CT was performed using a 128-slice CT scanner (SOMATOM Definition Flash; Siemens Medical Solutions, Erlangen, Germany) with the following protocol: detector collimation of 64 × 0.6 mm (equivalent to slice acquisition of 128 × 0.6 mm using the flying focal spot technique), table pitch adjusted to heart rate (0.17–0.38), rotation time of 275 ms, tube current–time product of 360 mAs, and tube voltage of 120-kVp, as previously described [15]. On coronary CTA analysis, we evaluated coronary artery segments with a diameter > 2 mm and defined plaque characteristics in accordance with the Society of Cardiovascular Computed Tomography [16]. Two experienced cardiovascular imaging researchers (K.O. and T.M.) interpreted the coronary CTA findings.

We defined positive remodelling as a remodelling index of > 1.1. Plaques with a CT attenuation number < 30 HU were defined as low-attenuation plaques. Spotty calcification was defined as a calcium burden length < 1.5 times the vessel diameter and a width less than two-thirds of the vessel diameter. The presence of two or more high-risk plaque features, including positive remodelling, low-attenuation plaques, and spotty calcification, indicated a high-risk plaque. Stenosis was significant if there was luminal narrowing of > 50% in any coronary artery. We defined adverse CTA findings as the presence of high-risk plaque and/or significant stenosis.

### Measurement of coronary artery calcium score and pericardial fat volume

We recorded the coronary artery calcium score (CACS) and pericardial fat volume based on the following parameters: 120 kVp, 150 mAs, and 3-mm thickness. All data were evaluated on a dedicated workstation (AZE Virtual Place; Canon medical systems Corporation, Otawara, Japan). The CACS was calculated using the Agatston method, which involved multiplying the area of each calcified plaque by a density factor determined using the peak pixel intensity within the plaque. The plaque-specific scores for all slices were added together [17]. The density factor was 1, 2, 3, and 4 for plaques with peak intensities of 130–199, 200–299, 300–399, and ≥ 400 Hounsfield units (HU), respectively. The pericardial fat volume was quantified by calculating the total volume of the tissues whose CT density ranged from − 190 to – 30 HU within the pericardium [18].

### Analysis of PCAT attenuation

**Fig. 2:**
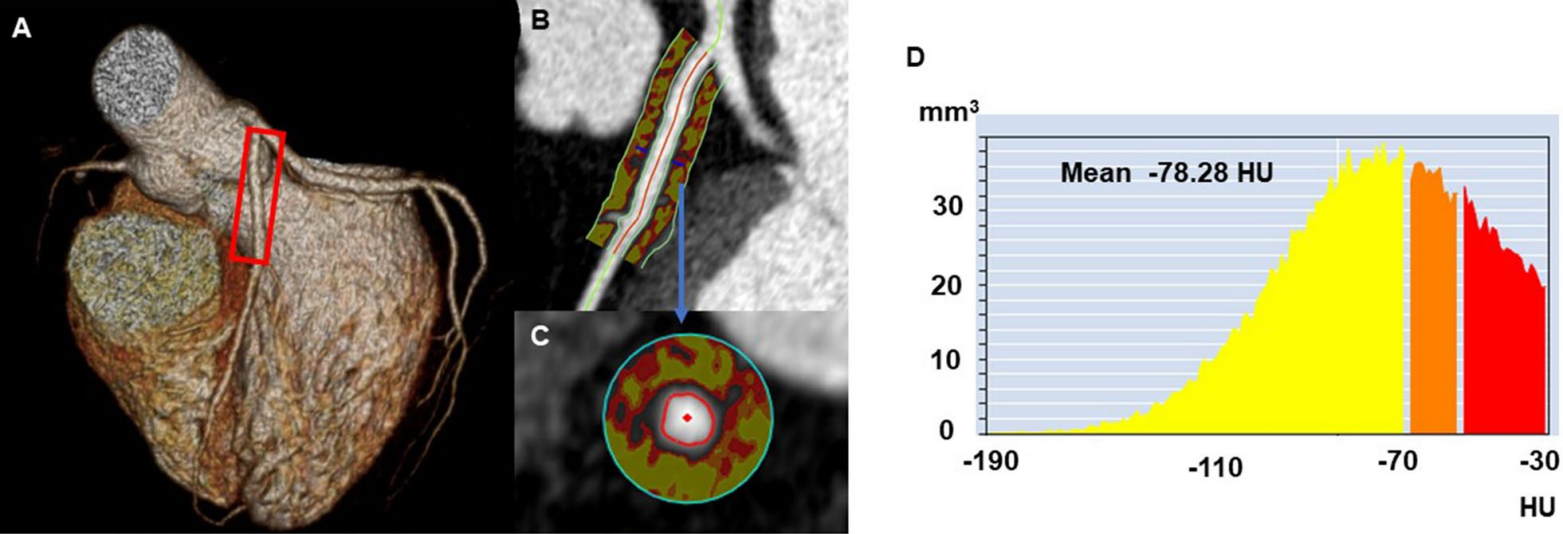
Representative case of pericoronary adipose tissue attenuation measured by coronary computed tomography (CT) angiography. Three-dimensional reconstruction of the heart (**A**); Pericoronary adipose tissue attenuation between − 190 and – 30 HU in the longitudinal view (**B**) and the cross-sectional view (**C**); around the proximal 40 mm of the left anterior descending artery; histogram of CT attenuation within the traced area (**D**)

In all patients, we measured the amount of PCAT attenuation using a dedicated workstation (Aquarius iNuition Edition version 4.4.13; TeraRecon Inc., Foster City, CA, USA). The selection of the LAD and RCA was based on a prior study that found an association between the PCAT attenuation of these two coronary arteries and cardiovascular events [10]. As shown in Fig. 2, we used an automated method to trace 40-mm proximal segments of the LAD and 10–50-mm proximal segments of the RCA while making manual adjustments to the automatic delineation of the coronary vessel wall. We could not extract the segmentations of 24 RCAs because of technical difficulties or non-dominant RCA.

PCAT was defined as the adipose tissue located within a radial distance from the outer vessel wall equal to the diameter of the coronary vessel [19], and adipose tissue was defined as all voxels with an attenuation between − 190 and – 30 HU. Based on this, the PCAT attenuation was automatically calculated as the mean CT attenuation value of PCAT. Two investigators (M.N. and T.N.) blinded to clinical and CT data performed the PCAT attenuation analysis. Intraobserver and interobserver agreement for the PCAT attenuation were very good [intraclass correlation coefficient: 0.921 (p < 0.001) and 0.951 (p < 0.001), respectively]. The final PCAT attenuation used for statistical analyses was the average value from the two investigators.

### Statistical analysis

Continuous variables are expressed as mean ± standard deviation or median with interquartile range, depending on the Shapiro–Wilks test for normality. Dichotomous variables are expressed as numbers (proportion). Differences in continuous variables between the two groups were analysed using the paired Student’s t-test or the Mann–Whitney U test, as appropriate. Categorical data were compared using a chi-square test or Fisher’s exact test, based on the category cell size.

In subsequent analysis, LAD-PCAT attenuation was used because LAD-PCAT attenuation was more closely associated with prognosis than RCA-PCAT attenuation in our study. Triglyceride data were log-transformed because they did not show a normal distribution. Similarly, because the distribution of the CACS was highly skewed, CACS was log-transformed after adding 1 to all calcium scores to manage values of 0 [log(CACS + 1)].

Univariate and multivariate linear regression analyses were performed to examine the predictors of LAD-PCAT attenuation. Cumulative survival estimates were calculated using the Kaplan–Meier method and compared using the log-rank test. Univariate Cox proportional hazard regression analyses were used to clarify variables associated with the occurrence of cardiovascular events, and the results were reported as hazard ratios (HRs) with 95% confidence intervals (CIs). To assess the independent prognostic value of clinical and imaging variables, multivariate Cox proportional hazard regression analyses were performed using backward selection and included variables with p < 0.10 in the univariate analysis.

The incremental value of LAD-PCAT attenuation compared to the adverse coronary CTA findings in predicting cardiovascular events was assessed using receiver operating characteristic (ROC) curve analysis and global chi-square test. The ROC curves were built based on a logistic regression model, and the Delong test was used to compare the C-statistics. All reported *p*-values were two-sided, and *p* < 0.05 was considered statistically significant. All statistical analyses were performed using the SPSS software (version 24; IBM Corp., Armonk, NY, USA) and the R statistical package (version 3.5.2; R Foundation for Statistical Computing, Vienna, Austria).

## Results

### Patient characteristics and coronary CTA findings

Our patients’ mean age was 66 ± 11 years, and 63% of them were male. The mean LAD and RCA-PCAT attenuation were − 70.6 ± 6.1 HU and − 68.0 ± 6.9 HU, respectively. The comparison of baseline characteristics and coronary CTA results between T2DM patients with and without cardiovascular events is shown in Table 1. We observed that patients with cardiovascular events had a higher Suita score (p = 0.001) and increased level of LDL-cholesterol (p = 0.013). Regarding CT findings, patients with cardiovascular events had higher LAD-PCAT attenuation (− 68.5 ± 6.5 HU vs. − 70.8 ± 6.1 HU, p = 0.045) and CACS (p = 0.003) and a higher prevalence of significant stenosis (p < 0.001) and high-risk plaques (p = 0.005). In contrast, RCA-PCAT attenuation was not significantly higher in patients with cardiovascular events than in those without cardiovascular events (− 66. 0 ± 8.2 HU vs. − 68.3 ± 6.7 HU, p = 0.089).

**Table 1:** Comparison of baseline characteristics between T2DM patients with and without cardiovascular events

Variable	All	Cardiovascular events		p value^a^
		Present	Absent	
n	333	31	302	
Age, years	66 ± 11	68 ± 10	66 ± 11	0.235
Male sex	211 (63)	21 (68)	190 (63)	0.595
Body mass index, kg/m^2^	25 ± 5	25 ± 5	25 ± 4	0.991
Hypertension	229 (69)	21 (68)	208 (69)	0.897
Dyslipidemia	219 (66)	20 (65)	199 (66)	0.878
Current Smoker	76 (23)	9 (29)	67 (22)	0.387
Obesity^b^	141 (42)	12 (39)	129 (43)	0.667
β-blocker	63 (19)	3 (10)	60 (20)	0.168
Calcium channel blocker	125 (38)	13 (42)	112 (37)	0.595
ACE-I or ARB	156 (47)	14 (45)	142 (47)	0.843
Statin	152 (46)	11 (36)	141 (47)	0.233
Insulin therapy	84 (25)	7 (23)	77 (26)	0.722
Oral antihyperglycemic drugs	184 (55)	16 (52)	168 (56)	0.668
Metformin	62 (19)	3 (10)	59 (20)	0.179
Alpha glucosidase inhibitor	52 (16)	2 (7)	50 (17)	0.140
DPP4 inhibitor	119 (36)	12 (39)	107 (35)	0.717
eGFR, mL/min/1.73 m^2^	68 ± 19	69 ± 21	68 ± 18	0.672
Total cholesterol, mg/dL	182 ± 32	190 ± 34	181 ± 32	0.145
LDL-cholesterol, mg/dL	107 ± 28	120 ± 35	106 ± 27	0.013
HDL-cholesterol, mg/dL	54 ± 17	53 ± 19	54 ± 16	0.666
Triglyceride, mg/dL	115 (85, 169)	114 (86, 169)	116 (84, 169)	0.859
HbA1c, %	7.4 ± 1.5	7.5 ± 1.2	7.4 ± 1.5	0.703
CACS	107 (2, 459)	298 (106, 998)	92 (0, 408)	0.003
Pericardial fat volume, mL	129 ± 53	133 ± 50	129 ± 54	0.633
LAD-PCAT attenuation, HU	− 70.6 ± 6.1	− 68.5 ± 6.5	− 70.8 ± 6.1	0.045
RCA-PCAT attenuation, HU	− 68.0 ± 6.9	− 66.0 ± 8.2	− 68.3 ± 6.7	0.089
Significant stenosis	135 (41)	22 (71)	113 (37)	< 0.001
High-risk plaque	81 (24)	14 (45)	67 (22)	0.005
Suita score	51 ± 10	55 ± 7	51 ± 11	0.001

Data are presented as mean ± standard deviation or number (%)

*T2DM* type 2 diabetes mellitus; *ACE-I* angiotensin-converting enzyme inhibitor; *ARB* angiotensin-receptor blocker; *DPP4* dipeptidyl peptidase-4; *eGFR* estimated glomerular filtration rate; *LAD* left anterior descending; *LDL* low-density lipoprotein; *HDL* high-density lipoprotein; *HbA1c* glycated hemoglobin A1c; *CACS* coronary artery calcium score; *PCAT* pericoronary adipose tissue; *RCA* right coronary artery; *HU* Hounsfield units

^a^Comparisons between patients with and without cardiovascular events

^b^Obesity was defined as body mass index ≥ 25 kg/m^2^

### Determinants of LAD-PCAT attenuation in T2DM patients

Univariate linear regression analysis revealed that the significant determinants of LAD-PCAT attenuation in T2DM patients were age (β = 0.059, p = 0.049), male sex (β = 3.879, p < 0.001), body mass index (β = − 0.241, p = 0.001), dyslipidaemia (β = − 2.577, p < 0.001), use of statins (β = − 2.104, p = 0.002), and a higher Suita score (β = 0.130, p < 0.001). All CT findings, including CACS, pericardial fat volume, significant stenosis, and high-risk plaques, were not associated with LAD-PCAT attenuation. Multivariate linear regression analysis revealed that the significant determinants of LAD-PCAT attenuation were male sex (β = 5.209, p < 0.001) and the use of statins (β = − 2.270, p = 0.024) (Additional file 1: Table S1).

### Association between LAD-PCAT attenuation and cardiovascular events in T2DM patients

**Fig. 3:**
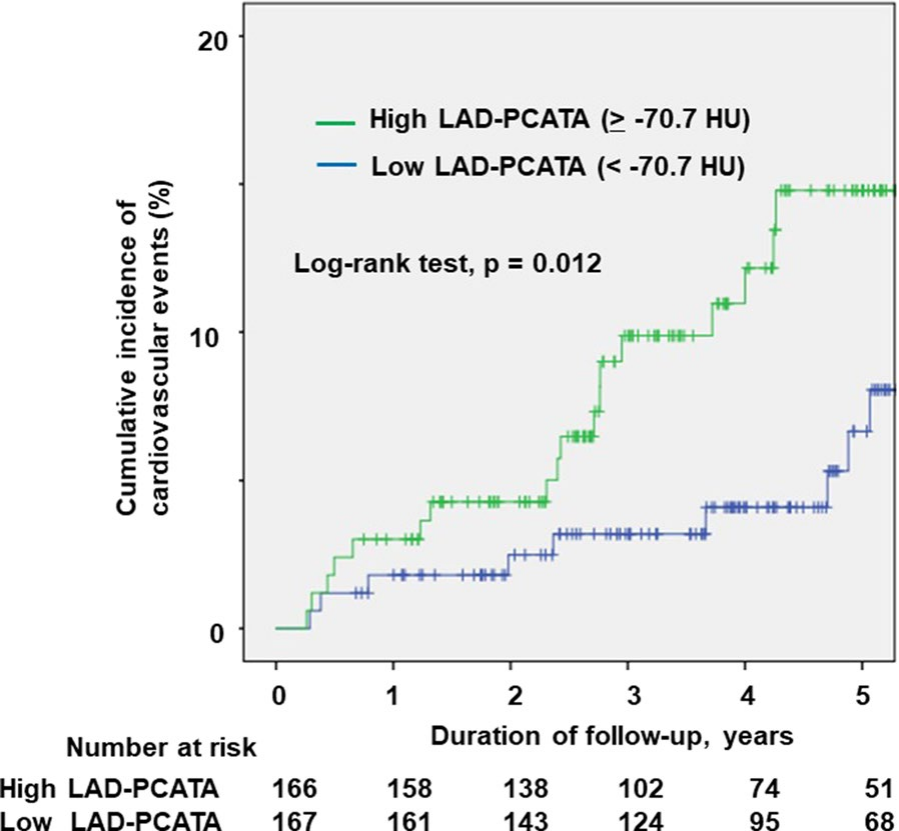
Kaplan–Meier curves of cumulative incidence of cardiovascular events. Kaplan–Meier curves according to low or high left anterior descending artery-pericoronary adipose tissue attenuation in patients with type 2 diabetes mellitus. *PCATA* pericoronary adipose tissue attenuation

We divided the patients into two groups based on median LAD-PCAT attenuation (− 70.7 HU). The comparison of baseline characteristics and coronary CTA results between the two groups is shown in Additional file 1: Table S2. During a median follow-up of 4 years, we recorded 31 cardiovascular events (2 cases of cardiovascular death, 14 of acute coronary syndrome, 4 of heart failure, and 11 late revascularisations) (Additional file 1: Table S3). Kaplan–Meier analysis showed that patients with high LAD-PCAT attenuation experienced more cardiovascular events than those with low LAD-PCAT attenuation during the follow-up (p = 0.012, log-rank test) (Fig. 3).

The univariate Cox regression analysis revealed that higher LDL-cholesterol, CACS, presence of significant stenosis and high-risk plaques, and high LAD-PCAT attenuation were associated with cardiovascular events. In the multivariate Cox regression analysis, we found that high LAD-PCAT attenuation was independently associated with cardiovascular events (HR = 2.689, 95% CI 1.166–6.199, p = 0.020) (Table 2).

**Table 2:** Factors associates with cardiovascular events

	Univariate		Multivariate	
	Hazard ratio (95% CI)	p value	Hazard ratio (95% CI)	p value
Age, years	1.029 (0.993–1.067)	0.119		
Male sex	1.346 (0.633–2.862)	0.440		
Body mass index, kg/m^2^	0.996 (0.920–1.078)	0.913		
Hypertension	0.975 (0.459–2.071)	0.947		
Dyslipidemia	0.931 (0.446–1.944)	0.850		
Current Smoker	1.570 (0.722–3.416)	0.441		
β-blocker	0.503 (0.152–1.662)	0.255		
Calcium channel blocker	1.507 (0.736–3.085)	0.262		
ACE-I or ARB	0.956 (0.471–1.939)	0.900		
Statin	0.669 (0.321–1.398)	0.285		
Insulin therapy	0.807 (0.348–1.876)	0.619		
Oral antihyperglycemic drugs	0.849 (0.420–1.719)	0.650		
Metformin	0.411 (0.125–1.353)	0.143		
Alpha glucosidase inhibitor	0.335 (0.080–1.406)	0.135		
DPP4 inhibitor	1.247 (0.603–2.578)	0.551		
eGFR, mL/min/1.73 m^2^	0.999 (0.979–1.019)	0.908		
Total cholesterol, mg/dL	1.006 (0.995–1.018)	0.273		
LDL-cholesterol, mg/dL	1.015 (1.002–1.029)	0.023	1.018 (1.005–1.032)	0.005
HDL-cholesterol, mg/dL	0.991 (0.968–1.015)	0.468		
Ln (triglyceride)	1.050 (0.531–2.078)	0.889		
HbA1c, %	1.029 (0.804–1.317)	0.813		
Ln (CACS + 1)	1.311 (1.109–1.549)	0.002		
Pericardial fat volume, mL	1.002 (0.996–1.008)	0.536		
Significant stenosis	4.750 (2.157–10.264)	< 0.001	4.286 (1.764–10.417)	0.001
High-risk plaque	2.850 (1.404–5.786)	0.004	2.076 (0.935–4.612)	0.073
High LAD-PCAT attenuation	2.562 (1.202–5.460)	0.015	2.689 (1.166–6.199)	0.026
Suita score	1.060 (1.019–1.102)	0.004		

*ACE-I* angiotensin-converting enzyme inhibitor; *ARB* angiotensin-receptor blocker; *DPP4* dipeptidyl peptidase-4; *eGFR* estimated glomerular filtration rate; *LDL* low-density lipoprotein; *HDL* high-density lipoprotein; *HbA1c* glycated hemoglobin A1c; *CACS* coronary artery calcium score; *LAD* left anterior descending artery; *PCAT* pericoronary adipose tissue

**Fig. 4:**
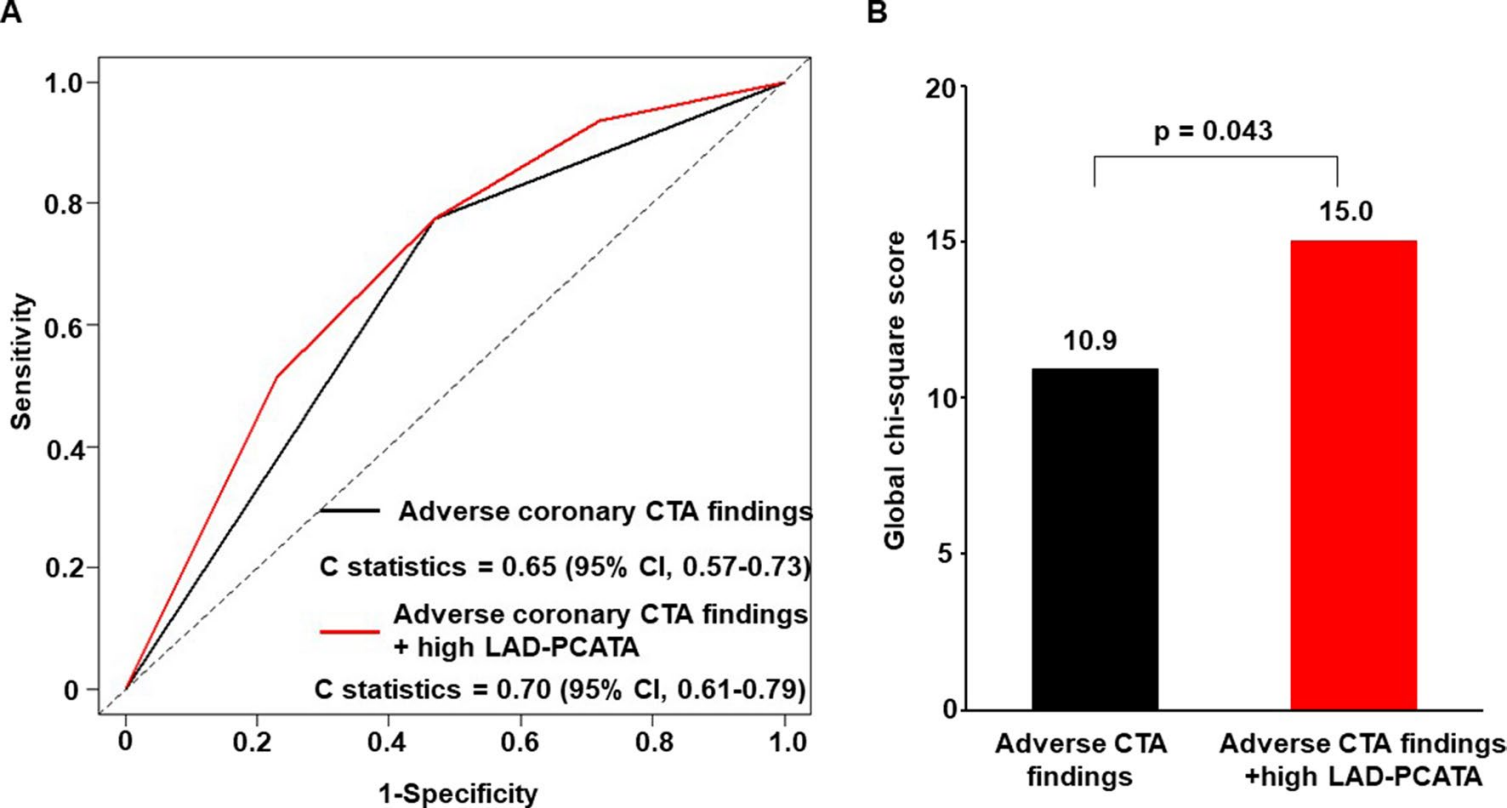
The incremental prognostic value of left anterior descending artery-pericoronary adipose tissue attenuation as compared with adverse coronary CTA findings for predicting cardiovascular events. **A** Receiver operating characteristic curves. **B** Global chi-square test. Adverse CTA finding, significant stenosis and/or high-risk plaque; *CTA* computed tomography angiography; *PCATA* pericoronary adipose tissue attenuation

Figure 4 shows the incremental value of adverse coronary CTA findings and LAD-PCAT attenuation in predicting risky cardiovascular events accurately. By considering high LAD-PCAT attenuation along with adverse coronary CTA findings, the C-statistic significantly increased from 0.65 to 0.70 (p = 0.037). Similarly, adding high LAD-PCAT attenuation to adverse coronary CTA findings significantly increased the global chi-square value from 10.9 to 15.0 (p = 0.043).

### Association of LAD-PCAT attenuation and cardiovascular events in T2DM patients with adverse CTA findings

We further investigated the impact of high LAD-PCAT attenuation on cardiovascular events in T2DM patients with adverse CTA findings, which includes significant stenosis and/or high-risk plaques (n = 166). During a median follow-up of 4.0 years, we recorded 24 cardiovascular events (1 case of cardiovascular death, 11 of acute coronary syndrome, 2 of heart failure, and 10 of late revascularisation). In this subgroup analysis, the median value of LAD-PCAT attenuation was − 70.2 HU Patients with high LAD-PCAT attenuation (≥ − 70.2 HU) experienced more cardiovascular events than those with low PCAT attenuation during the follow-up (p = 0.024, log-rank test). Cox univariate regression analysis showed that high LAD-PCAT attenuation was significantly associated with cardiovascular events (HR = 2.536, 95% CI 1.070–6.009, p = 0.034). In the multivariate Cox regression analysis, high LAD-PCAT attenuation was independently associated with cardiovascular events (HR = 2.461, 95% CI 1.034–5.855, p = 0.042) (Additional file 1: Table S4).

## Discussion

To our best knowledge, this is the first study to demonstrate that increased pericoronary inflammation is significantly associated with cardiovascular events in T2DM patients. Our findings show that LAD-PCAT attenuation along with the adverse coronary CTA findings (significant stenosis and/or high-risk plaques) improved the model fit for predicting cardiovascular events in T2DM patients. Our findings may help physicians identify T2DM patients at a higher risk of developing cardiovascular events.

In the previous CRISP-CT study, increased LAD and RCA-PCAT attenuation were related to a risk of cardiovascular events in individuals undergoing clinically indicated diagnostic coronary CTA [10]. However, it was unknown whether this result could be applied directly to T2DM patients at a considerably higher cardiovascular risk than individuals in the CRIPS-CT study. T2DM patients have a higher chronic inflammatory status than non-T2DM patients [20] and exhibit a higher rate and greater proportion of adverse plaque progression [21]. Our study confirmed that high LAD-PCAT attenuation was associated with increased cardiovascular risk in T2DM patients, independent of the status of their coronary plaque. These results suggest that PCAT attenuation and adverse CTA findings affect cardiovascular events differently in T2DM patients.

Yu et al. recently reported that RCA-PCAT attenuation, and not LAD-PCAT attenuation, was significantly higher in T2DM patients, thereby suggesting that RCA-PCAT attenuation is a more reproducible measurement of global inflammatory status compared to LAD-PCAT attenuation [22]. In addition, the recent study by van Diemen et al. reported that RCA-PCAT attenuation had more superior prognostic value than LAD-PCAT attenuation [23]. Of importance, RCA-PCAT attenuation in patients with cardiovascular events was higher than that in patients without cardiovascular events, but the difference was not statistically significant in our study. There are some possible explanations for this discordance with previous reports. Firstly, the power of our study is hampered by a lower study population and lower cardiovascular events compared to previous studies. Secondly, PCAT attenuation values are known to be affected by various factors, such as CT scanners and scan parameters [24]. Although our acquisition parameters were unified in all patients in this study, some unknown factors may have affected the PCAT attenuation values. Finally, previous studies mostly included a Caucasian population, whereas our study comprised only Japanese patients. The ethnic differences might have influenced the association between PCAT attenuation and cardiovascular outcomes. Further studies are needed to clarify the factors affecting PCAT attenuation and its prognostic value in various populations.

T2DM patients typically have more adverse CTA findings than those without T2DM. Although adverse coronary CTA findings, such as significant stenosis and high-risk plaques, are established risk factors for cardiovascular events in T2DM patients [8, 25], their positive predictive value to identify subsequent cardiovascular events remains limited. A recent cohort study of T2DM patients reported that only 3.5% of coronary lesions with coronary CTA-detected stenotic sites (≥ 50%) and high-risk plaques later caused cardiovascular incidents [8], which means that 96% of lesions with these CTA findings remained event-free. The formation of plaque morphology is a dynamic process. Plaques can gain or lose these plaque features over time [26], which limits their diagnostic capability. Increased PCAT attenuation is independently associated with total plaque and high-risk plaque progression [27]; however, in this study, LAD-PCAT attenuation was not associated with significant stenosis and high-risk plaques. Given these varied findings, we believe that PCAT attenuation might have a different impact on cardiovascular events compared with adverse CTA findings.

A previous cohort study reported that adding PCAT attenuation to coronary CTA findings enhanced its predictive value in patients undergoing clinically coronary CTA [28]. Moreover, a recent study using machine learning reported that the radiomic signature of PCAT attenuation provides additional risk stratification [29]. In this study, we confirmed the incremental prognostic value of LAD-PCAT attenuation in T2DM patients. Adverse CTA findings represent a series of structural changes that appear later in the inflammatory process. Contrarily, PCAT attenuation represents the change in intracellular lipid accumulation caused by early and chronic inflammation [28]. Thus, combining PCAT attenuation and CTA-detectable plaque features could provide important complementary information on the disease status of coronary arteries.

PCAT attenuation is not a static value; instead, it appears to be modifiable by applying medical treatment. Previous studies have reported that PCAT can markedly reduce in response to anti-inflammatory interventions and statin therapy [19, 30]. However, further studies are needed to validate the effects of these medical therapies on PCAT attenuation and their clinical outcomes in T2DM patients.

In this study, cardiovascular events included hospitalisation for heart failure. Emerging evidence indicates that coronary microvascular dysfunction is linked to heart failure with preserved ejection fraction [31, 32]. Meanwhile, recent studies have demonstrated the significant association between PCAT attenuation and microvascular coronary dysfunction [33, 34]. Thus, there is a possibility that high PCAT attenuation is associated with an increased risk of heart failure. In the current study, all heart failure events were documented in patients with high PCAT attenuation. However, we could not conclude its significant association due to the small number of heart failure events. Future studies are needed to confirm the relationship between PCAT attenuation and heart failure.

This study had some limitations. First, this was a single-centre study, and the number of cardiovascular events that occurred was relatively small. The study endpoint was decided using soft events, such as late revascularisation. Second, we have no data about the duration of T2DM and longitudinal information on changes in the patients’ medications. Third, since our study started in 2011, our cohort data did not contain information about sodium-glucose cotransporter-2 inhibitors and glucagon-like peptide-1 receptor agonists, both of which are now known to be effective in reducing cardiovascular events [35, 36]. Future studies are required to evaluate whether these medications effectively reduce PCAT attenuation. Finally, information on longitudinal medication, risk factor control, body mass index changes, and lifestyle fluctuations during the follow-up period was not available.

## Conclusions

Our study demonstrated that high LAD-PCAT attenuation on coronary CTA was significantly associated with cardiovascular events and had an incremental prognostic value in reducing their risk in T2DM patients. Our findings may help physicians and healthcare workers promptly identify T2DM patients at higher risk for future cardiovascular events and administer corrective therapies accordingly.

## Supplementary Information

**Additional file 1: Table S1.** Determinants of pericoronary adipose tissue attenuation in the left anterior descending artery. **Table S2. **Comparison of baseline characteristics between patients with high and low pericoronary adipose tissue attenuation in the left anterior descending artery. **Table S3.** Cardiovascular events in T2DM patients with high and low pericoronary adipose tissue attenuation in the left anterior descending artery. **Table S4. **Factors associated with cardiovascular events in patients with adverse coronary computed tomography angiography findings.

## Data Availability

The datasets used and/or analyzed during the current study are available from the corresponding author on reasonable request.

## Abbreviations

ACR: American College of Radiology
CACS: Coronary artery calcium score
CAD: Coronary artery disease
CI: Confidence interval
CT: Computed tomography
CTA: Computed tomography angiography
CRISP-CT: Cardiovascular RISk Prediction using Computed Tomography
HR: Hazard ratio
HU: Hounsfield units
LAD: Left anterior descending
LDL: Low-density lipoprotein
NASCI: North American Society for Cardiovascular Imaging
PCAT: Pericoronary adipose tissue
RCA: Right coronary artery
ROC: Receiver operating characteristic
SCCT: Society of Cardiovascular Computed Tomography
T2DM: Type 2 diabetes mellitus
