# Supplementary material for: High pericoronary adipose tissue attenuation on computed tomography angiography predicts cardiovascular events in patients with type 2 diabetes mellitus: post-hoc analysis from a prospective cohort study
Source: Cardiovasc Diabetol. 2022 Mar 18;21:44. doi: 10.1186/s12933-022-01478-9 (PMC8933955; doi:10.1186/s12933-022-01478-9)
Supplement: Supplementary file 1 — Additional file 1: Table S1. Determinants of pericoronary adipose tissue attenuation in the left anterior descending artery. Table S2. Comparison of baseline characteristics between patients with high and low pericoronary adipose tissue attenuation in the left anterior descending artery. Table S3. Cardiovascular events in T2DM patients with high and low pericoronary adipose tissue attenuation in the left anterior descending artery. Table S4. Factors associated with cardiovascular events in patients with adverse coronary computed tomography angiography findings. [file 12933_2022_1478_MOESM1_ESM.docx]

**Table S1.** Determinants of pericoronary adipose tissue attenuation in the left anterior descending artery

|  | Univariate | | Multivariate | | |
| --- | --- | --- | --- | --- | --- |
|  | β coefficient (95%CI) | p-value | | β coefficient (95%CI) | p-value |
| Age, years | 0.059 (0.000- 0.117) | 0.049 | | 0.048 (-0.010, 0.106) | 0.105 |
| Male sex | 3.879(2.571- 5.188) | <0.001 | | 5.209(2.182, 4.831) | <0.001 |
| Body mass index, kg/m^2^ | -0.241(-0.386- -0.097) | 0.001 | | -0.094(-0.245- -0.057) | 0.222 |
| Hypertension | -0.432(-1.860- -0.996) | 0.552 | |  |  |
| Dyslipidaemia | -2.577(-3.944- -1.210) | <0.001 | |  |  |
| Current smoker | 0.686(-0.890- 2.262) | 0.392 | |  |  |
| β-blocker | 0.397(-1.293- 2.087) | 0.645 | |  |  |
| Calcium channel blocker | -0.057(-1.425- 1.310) | 0.934 | |  |  |
| ACE-I or ARB | -0.274(-1.601- 1.052) | 0.684 | |  |  |
| Statin | -2.104(-3.414- -0.794) | 0.002 | | -1.487(-2.775- -0.198) | 0.024 |
| Insulin therapy | -1.163(-2.682- 0.356) | 0.133 | |  |  |
| Oral antihyperglycemic drugs | 0.465(-0.866- 1.795) | 0.493 | |  |  |
| Metformin | -1.426(-3.120- 0.268) | 0.099 | |  |  |
| Alpha glucosidase inhibitor | 0.927(-0.894- 2.748) | 0.318 | |  |  |
| DPP4 inhibitors | 0.782(-0.597- 2.161) | 0.266 | |  |  |
| eGFR, mL/min/1.73 m^2^ | -0.005(-0.042- 0.032) | 0.790 | |  |  |
| Total cholesterol, mg/dL | 0.005(-0.017- 0.027) | 0.649 | |  |  |
| LDL-cholesterol, mg/dL | 0.010(-0.015- 0.036) | 0.428 | |  |  |
| HDL-cholesterol, mg/dL | -0.001(-0.044- 0.042) | 0.980 | |  |  |
| Ln (triglyceride) | 0.190(-2.194- 0.437) | 0.190 | |  |  |
| HbA1c, % | -0.347(-0.827- 0.133) | 0.155 | |  |  |
| Ln (CACS+1) | 0.168(-0.079- 0.415) | 0.181 | |  |  |
| Pericardial fat volume, mL | -0.006(-0.018- 0.007) | 0.373 | |  |  |
| Significant stenosis | 0.880(-0.466- 2.225) | 0.199 | |  |  |
| High-risk plaque | -0.042(-1.585- 1.502) | 0.958 | |  |  |
| Suita score | 0.130 (0.067- 0.193) | <0.001 | |  |  |

ACE-I, angiotensin-converting enzyme inhibitor; ARB, angiotensin-receptor blocker; DPP4, dipeptidyl peptidase-4; eGFR, estimated glomerular filtration rate; LDL, low-density lipoprotein; HDL, high-density lipoprotein; HbA1c, glycated haemoglobin A1c; CACS, coronary artery calcium score; PCAT, pericoronary adipose tissue.

**Table S2.** Comparison of baseline characteristics between patients with high and low pericoronary adipose tissue attenuation in the left anterior descending artery

|  | All | High LAD-PCAT attenuation | Low LAD-PCAT attenuation | p-value |
| --- | --- | --- | --- | --- |
| n | 333 | 166 | 167 |  |
| Age, years | 66 ± 11 | 67 ± 11 | 65 ± 11 | 0.100 |
| Male sex | 211 (63) | 127 (77) | 84 (50) | <0.001 |
| Body mass index, kg/m^2^ | 25 ± 5 | 24 ± 4 | 26 ± 5 | 0.011 |
| Hypertension | 229 (69) | 112 (68) | 117 (70) | 0.610 |
| Dyslipidaemia | 219 (66) | 100 (60) | 119 (71) | 0.034 |
| Current Smoker | 76 (23) | 43 (26) | 33 (20) | 0.182 |
| Obesity^a^ | 141 (42) | 58 (35) | 83 (50) | 0.006 |
| β-blocker | 63 (19) | 35 (21) | 28 (17) | 0.314 |
| Calcium channel blocker | 125 (38) | 65 (39) | 60 (36) | 0.543 |
| ACE-I or ARB | 156 (47) | 77 (46) | 79 (47) | 0.866 |
| Statin | 152 (46) | 67 (40) | 85 (51) | 0.054 |
| eGFR, ml/min/1.73m^2^ | 68 ± 19 | 68 ± 20 | 68 ± 17 | 0.932 |
| Total cholesterol, mg/dl | 182 ± 32 | 181 ± 32 | 182 ± 32 | 0.845 |
| LDL-cholesterol, mg/dl | 107 ± 28 | 108 ± 30 | 107 ± 27 | 0.692 |
| HDL-cholesterol, mg/dl | 54 ± 17 | 54 ± 16 | 54 ± 17 | 0.943 |
| Triglyceride, mg/dl | 115(85, 169) | 118 (83, 170) | 114 (85, 169) | 0.772 |
| HbA1c, % | 7.4 ± 1.5 | 7.3 ± 1.4 | 7.5 ± 1.5 | 0.284 |
| CACS | 107 (2, 459) | 177 (12, 555) | 60 (0, 339) | 0.015 |
| Pericardial fat volume, mL | 120 (92, 158) | 120 (92, 157) | 120 (90, 162) | 0.657 |
| LAD-PCAT attenuation, HU | -70.6 ± 6.1 | -67.4 ± 6.4 | -70.8 ± 6.1 | 0.016 |
| Significant stenosis | 135 (41) | 74 (45) | 61 (37) | 0.135 |
| High-risk plaque | 81 (24) | 39 (24) | 42 (25) | 0.725 |
| Suita score | 51 ± 10 | 53 ± 10 | 49 ± 10 | 0.002 |

^a^Obesity was defined as body mass index > 25 kg/m^2^.

ACE-I, angiotensin-converting enzyme inhibitor; ARB, angiotensin-receptor blocker; DPP4, dipeptidyl peptidase-4; eGFR, estimated glomerular filtration rate; LDL, low-density lipoprotein; HDL, high-density lipoprotein; HbA1c, glycated haemoglobin A1c; CACS, coronary artery calcium score; LAD, left anterior descending artery; PCAT, pericoronary adipose tissue; HU, Hounsfield units.

**Table S3.** Cardiovascular events in T2DM patients with high and low pericoronary adipose tissue attenuation in the left anterior descending artery

|  | All (n=333) | high PCAT attenuation (n=166) | low PCAT attenuation (n=167) |
| --- | --- | --- | --- |
| Composite cardiovascular events | 31 (9.3) | 21 (12.7) | 10 (6.0) |
| Cardiovascular death | 2 (0.6) | 2 (1.2) | none |
| Acute coronary syndrome | 14 (4.2) | 8 (4.8) | 6 (3.6) |
| Heart failure | 4 (1.2) | 4 (2.4) | none |
| Late revascularisations | 11 (3.3) | 7 (4.2) | 4 (2.4) |

Data are presented as number (%)

T2DM, Type 2 diabetes mellitus; PCAT, pericoronary adipose tissue.

**Table S4.** Factors associated with cardiovascular events in patients with adverse coronary computed tomography angiography findings

|  | Univariate | | Multivariate | |
| --- | --- | --- | --- | --- |
|  | Hazard ratio(95%CI) | p-value | Hazard ratio(95%CI) | p-value |
| Age, years | 1.008 (0.962–1.056) | 0.964 |  |  |
| Male sex | 1.092 (0.452–2.638) | 0.846 |  |  |
| Body mass index, kg/m^2^ | 1.056 (0.968–1.152) | 0.219 |  |  |
| Hypertension | 1.325 (0.495–3.550) | 0.576 |  |  |
| Dyslipidaemia | 0.820 (0.350–1.917) | 0.646 |  |  |
| Current Smoker | 1.643 (0.674–4.003) | 0.275 |  |  |
| β-blocker | 0.574 (0.171–1.927) | 0.369 |  |  |
| Calcium channel blocker | 2.415 (1.078–5.407) | 0.032 | 2.334 (1.041–5.233) | 0.040 |
| ACE-I or ARB | 1.137 (0.509–2.541) | 0.882 |  |  |
| Statin | 0.529 (0.231–1.210) | 0.131 |  |  |
| Insulin therapy | 0.442 (0.150–1.300) | 0.138 |  |  |
| Oral antihyperglycemic drugs | 0.597 (0.268–1.330) | 0.207 |  |  |
| Metformin | 0.154 (0.021–1.142) | 0.067 |  |  |
| Alpha glucosidase inhibitor | 0.145 (0.020–1.078) | 0.059 |  |  |
| DPP4 inhibitor | 1.105 (0.480–2.544) | 0.814 |  |  |
| eGFR, ml/min/1.73m^2^ | 1.009 (0.985–1.034) | 0.467 |  |  |
| Total cholesterol, mg/dl | 1.009 (0.995–1.022) | 0.202 |  |  |
| LDL-cholesterol, mg/dl | 1.015 (1.000–1.030) | 0.057 |  |  |
| HDL-cholesterol, mg/dl | 1.001 (0.974–1.028) | 0.963 |  |  |
| Ln (triglyceride) | 1.000 (0.463–2.163) | 0.999 |  |  |
| HbA1c | 0.908 (0.638–1.295) | 0.595 |  |  |
| Ln (CACS+1) | 1.074 (0.841–1.371) | 0.565 |  |  |
| Pericardial fat volume, mL | 1.003 (0.997–1.010) | 0.320 |  |  |
| LAD-High PCAT attenuation | 2.536 (1.070–6.009) | 0.034 | 2.461 (1.034–5.855) | 0.042 |
| Suita score | 1.042 (0.993–1.093) | 0.094 |  |  |

ACE-I, angiotensin-converting enzyme inhibitor; ARB, angiotensin-receptor blocker; DPP4, dipeptidyl peptidase-4; eGFR, estimated glomerular filtration rate; LDL, low-density lipoprotein; HDL, high-density lipoprotein; HbA1c, glycated haemoglobin A1c; CACS, coronary artery calcium score; LAD, left anterior descending artery; PCAT, pericoronary adipose tissue.
